# Neo-Aplysiatoxin A Isolated from Okinawan Cyanobacterium *Moorea Producens*

**DOI:** 10.3390/molecules25030457

**Published:** 2020-01-22

**Authors:** Mioko Kawaguchi, Masayuki Satake, Bo-Tao Zhang, Yue-Yun Xiao, Masayuki Fukuoka, Hajime Uchida, Hiroshi Nagai

**Affiliations:** 1 Department of Ocean Sciences, Tokyo University of Marine Science and Technology, Tokyo 108-8477, Japan; ballet305.fouette@gmail.com (M.K.); zjsmyy1200@gmail.com (B.-T.Z.); xyy1993123@gmail.com (Y.-Y.X.); 0917mfukuoka@gmail.com (M.F.); 2 Department of Chemistry, The University of Tokyo, Tokyo 113-0033, Japan; msatake@chem.s.u-tokyo.ac.jp; 3 National Research Institute of Fisheries Science, Japan Fisheries Research and Education Agency, Yokohama 236-8648, Japan; huchida@affrc.go.jp

**Keywords:** cyanobacteria, aplysiatoxin, *Moorea producens*, cytotoxicity, bioactivity

## Abstract

A new aplysiatoxin derivative, neo-aplysiatoxin A (**1**), along with seven known compounds, neo-debromoaplysiatoxin A (**2**), dolastatin 3 (**3**), lyngbic acid (**4**), malyngamide M (**5**), hermitamide A (**6**), (−)-loliolide (**7**), and (+)-epiloliolide (**8**), was isolated from the Okinawan cyanobacterium *Moorea producens*. Their structures were elucidated on the basis of spectroscopic data, including high-resolution mass spectrometry and nuclear magnetic resonance. The compounds were evaluated for cytotoxic and diatom growth inhibition activities.

## 1. Introduction

**Figure 1:**
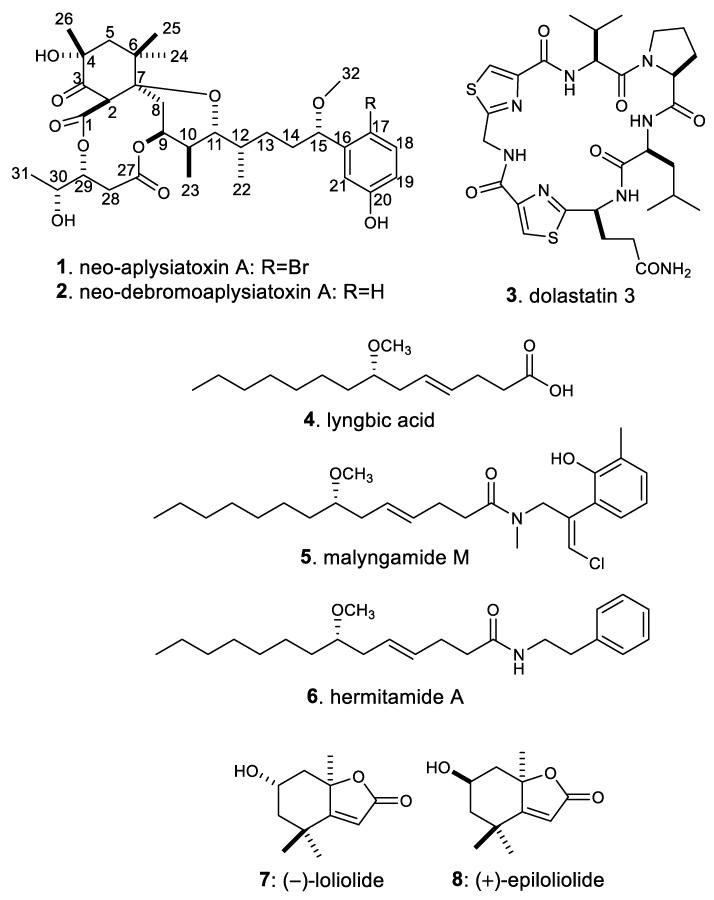
Structures of compounds **1** to **8**.

In July of 2010, an outbreak of the marine cyanobacterium *Moorea producens* occurred in Okinawa, Japan. The sample used in this study was collected at that time and at that site. *M. producens* (formerly *Lyngbya majuscula*) is known as a producer of various toxic compounds that affect human health [1,2,3]. Previously, we initiated the analysis of toxic constituents in this sample. Our previous studies revealed that this sample contained aplysiatoxin and related compounds [4,5]. It is known that aplysiatoxins strongly potentiate protein kinase C (PKC) activity and that PKC activation by aplysiatoxins causes potent tumor-promoting actions [6,7,8,9,10]. The regulation of PKC activity has been shown to be a valuable developmental method for obtaining anti-cancer drugs [11,12]. In fact, simplified aplysiatoxin derivatives were shown to have anti-cancer activity [13,14]. Recently, it was revealed that neo-debromoaplysiatoxin (**2**) had an inhibitory effect on K^+^ channel activity, suggesting that their potential bioactivity could make aplysiatoxin derivatives an intriguing research subject [15]. As such, we continued to purify the compounds extracted from *M. producens.* Finally, the new aplysiatoxin derivative neo-aplysiatoxin A (**1**), along with seven known compounds (**2**–**8**, Figure 1), was isolated [15,16,17,18,19,20]. In this report, the isolation, structure elucidation, and bioactivities of these compounds are presented.

## 2. Results and Discussion

### 2.1. Structure Elucidation of the Compounds

**Figure 2:**
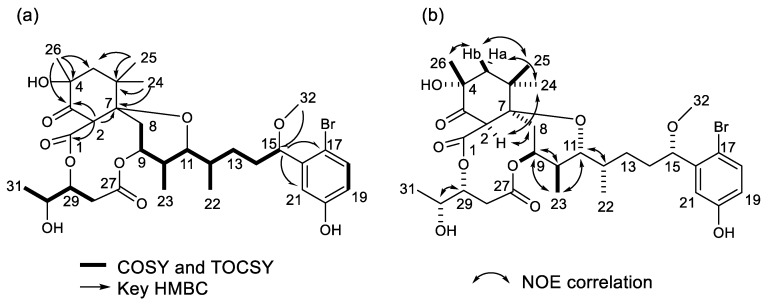
(**a**) Observed COSY, TOCSY, and HMBC correlations of **1**, (**b**) NOE correlations of **1**.

Compound **1** was isolated as a colorless solid ([α]_D_^17^ + 16 (c 0.054, MeOH)). The nuclear magnetic resonance (^1^H NMR) spectrum showed that it was an aplysiatoxin-related compound. The molecular formula of **1** was deduced to be C_32_H_45_BrO_10_ ([M−H]^−^ 667.2069 and 669.2047, calculated. 667.2112, and 669.2098, Figure S1), suggesting that it contained a bromophenol side chain. This was confirmed by the presence of a maximum at 281 nm (log ε 3.716) in the UV spectrum. The HSQC spectra (Figure S5) of **1** showed seven methyl groups (three singlets, three doublets, and a methoxy), five methylenes, two methines bonded to methyls, and a methine in the aliphatic region, as well as five oxygenated methines and three aromatic protons in the bromophenol side chain. Nine quaternary carbons (one aliphatic, two oxygenated, three aromatics in the bromophenol, two esters, and one ketone) were observed (Table 1). A ketone observed at δ_C_ 203.3 in methanol-*d*_4_ and a singlet methine (H-2) at δ_H_ 4.47, and HMBC correlations (Figure S6) from H-2 and Me-26 (δ_H_ 1.27) to the ketone (C-3) and from H-2 and two singlet methyls (Me-24 and Me-25) to an oxygenated quaternary carbon (C-7) at δ_C_ 83.1 suggested that **1** had a trimethylcyclohexanone structure analogous to 30-methyloscillatoxin D and neo-debromoaplysiatoxin A [4,15]. The partial structures H_2_-8 to H-9, H-10 (Me-23) to H-12 (Me-22), H_2_-14 to H-15, H-18 to H-19, and H_2_-28 to Me-31 were elucidated via ^1^H-^1^H COSY analysis (Figure 2a). 

As seen with neo-debromoaplysiatoxin A (**2**) [15], an HMBC correlation (Figure 2a) from Me-26 to an oxygenated quaternary carbon at δ_C_ 74.4 indicated that a hydroxy group was attached to C-4 in the trimethylcyclohexanone. The proton chemical shifts of H_2_-8 (δ_H_ 2.05 and 2.19) and H-9 (δ_H_ 5.01) corresponded to a methylene group and an oxymethine group, respectively, indicating the absence of a double bond in a 6-membered ether ring, which has previously been observed in oscillatoxin D analogs [4]. HMBC correlations observed from H-2 to C-1, from H_2_-5 to C-4 and C-7, from H_2_-14 to C-12 and C-13, and from H-15 to C-17 and C-21 connected the partial structures from C-1 to C-26, and HMBC correlations from H_2_-28 to C-27 confirmed a pentanoate structure. The methoxy (Me-32) proton at δ_H_ 3.27 was confirmed by the HMBC spectrum to be bonded to C-15. Although HMBC correlations from H-9 to C-27 and H-29 to C-1 were not observed, the proton chemical shifts of H-9 at δ_H_ 5.01 and of H-29 at δ_H_ 5.10 suggested ester linkages between C-9 and C-27 and C-1 and C-29 respectively. These results suggested that **1** was a 17-brominated analog of neo-debromoaplysiatoxin A (**2**). The configuration of **1** was deduced by comparison of its proton chemical shifts with those of neo-debromoaplysiatoxin A (**2**), whose structure was confirmed by X-ray analysis [15]. The configuration at C-2, C-4, and C-7 was deduced from NOE correlations (Figure 2b, Figure S7) and the proton chemical shifts. The observed NOE correlations Me-25/H-5b and Me-26/H-5b suggested Me-25 and Me-26 resided in the same direction. The NOE correlations H-2/Me-24 and H-2/H8b indicated H-2, H_2_-8, and Me-24 positioned the α-orientation on the cyclohexanone. The proton chemical shifts of H-2, H_2_-5, H_2_-8, and Me-26 were in accordance with those of neo-debromoaplysiatoxin A [15]. These NOE correlations and proton chemical shifts indicated the configuration of the cyclohexanone shown in Figure 1. The proton coupling constants of 4.5 Hz for H-8a/H-9 and 4.5 Hz for H-8b/H-9, and NOE correlations H-9/H-10 and H-9/Me-23 indicated an equatorial orientation of H-9 on a 6-member ether ring. The large coupling constant (*J* = 10.8 Hz) of H-10/H-11 indicated that both H-10 and H-11 were axial protons. The small coupling constant (*J* = 1.7 Hz) and the NOE correlation H-11/H-12 suggested a gauche conformer of H-11/H-12 analogous with neo-debromoaplysiatoxin A. The NOE correlation H-29/H-30 and the proton coupling constant (*J* = 5.2 Hz) suggested the stereochemistry of H-29 and H-30 was syn. The proton chemical shift and the coupling constants (*J* = 7.5, 4.5 Hz) of H-15 were identical with those of aplysiatoxins possessing a bromophenol sidechain [4]. Therefore, the configuration at C-15 in **1** was deduced to be the same as that of aplysiatoxins. These analyses revealed **1** to be a 17-brominated analog of neo-debromoaplysiatoxin A (**2**). Therefore, we designated **1** as neo-aplysiatoxin A.

Seven known compounds were identified as neo-debromoaplysiatoxin A (**2**) [15], dolastatin 3 (**3**) [16], lyngbic acid (**4**) [17], malyngamide M (**5**) [18], hermitamide A (**6**) [19], (−)-loliolide (**7**) [20], and (+)-epiloliolide (**8**) [20] through comparison of their spectroscopic data with those reported in the literature. Compounds **2**–**4** and **6** were formerly reported from cyanobacteria [15,16,17,19]. Malyngamide M (**5**) had been isolated from a Hawaiian red alga [18]. However, it has been suggested that the true producer of malyngamide M (**5**) is a cyanobacterium growing epiphytically on the red alga [18]. Our study strongly supports this hypothesis. Loliolides **7** and **8** are norisoprenoids reported mainly from terrestrial higher plants and marine macroalgae [21]. This study represents the first occurrence of loliolides **7** and **8** isolated from cyanobacteria. These findings suggest that cyanobacteria are the true origins of loliolides isolated from marine animals such as sponges [22,23,24] and mollusks [25,26]. These results indicate that cyanobacteria may be the producers of many marine-originated compounds with unknown origins. The wide variety of compounds obtained in this study, in addition to the former studies on this cyanobacterium sample [4,5], highlighted once again that cyanobacterium *Moorea producens* is a rich source of unique secondary metabolites.

### 2.2. Biological Activities

These compounds were tested for cytotoxicity against L1210 mouse lymphoma cells and growth inhibition activity against a marine diatom *Nitzchia amabilis*. The results of the bioactivity tests are shown in Table 2. Neo-aplysiatoxin A (**1**) showed the same biotoxicities as neo-debromoaplysiatoxin A (**2**) at the tested doses (10 µg/mL). The bioactive potencies of **1** and **2** positioned them as the most active compounds when compared to the activities of aplysiatoxin and its related compounds in the same bioactivity tests [4,5]. It was previously reported that neo-debromoaplysiatoxin A (**2**) showed K^+^ channel inhibition activity [15]. Therefore, neo-aplysiatoxin A (**1**), which is closely related, could also be a K^+^ channel inhibitor. The mode of action of neo-aplysiatoxin A (**1**) is an intriguing research subject.

Dolastatin 3 (**3**) had been reported as an anticancer drug lead with its potent cytotoxicity [27,28], however in this study dolastatin 3 (**3**) showed no cytotoxicity against the L1210 cell line at a concentration of 10 µg/mL. Furthermore, in previous studies, synthetic dolastatin 3 also failed to show cytotoxicity [29]. Therefore, the cytotoxic activity of dolastatin 3 should be reconsidered.

Malyngamide M (**5**) and hermitamide A (**6**) both exhibited biotoxicity. In previous studies, malyngamide M (**5**) and hermitamide A (**6**) showed weak cytotoxicity against mouse neuroblastoma cells [18] and mild ichthyotoxic activity to goldfish [19] respectively.

Lyngbic acid (**4**), (−)-loliolide (**7**), and (+)-epiloliolide (**8**) showed no toxicity at the tested dose in this study.

## 3. Materials and Methods

### 3.1. General Experimental Procedure

HPLC was performed using a Hitachi Chromaster HPLC System (Hitachi High-Tech Science Co., Tokyo, Japan). HR-ESI-MS spectral data were collected using a Bruker micrOTOF QII (Bruker Co., Bremen, Germany) mass spectrometer. NMR spectra were recorded in methanol-*d*_4_ using a Bruker AVANCE III 600 spectrometer. Optical rotations were measured using a JASCO P-2100 (JASCO Co., Tokyo, Japan) with a 10 mm length cell. UV spectra were measured using a JASCO V-550 UV-spectrometer (JASCO Co., Tokyo, Japan). Bioassay results were recorded on a Model 550 microplate reader (Bio-Rad, Hercules, CA, USA).

### 3.2. Marine Cyanobacterium M. Producens

Samples of the marine cyanobacterium *Moorea producens* were collected from Kuba Beach, Nakagusuku, Okinawa, Japan, in July 2010. After freeze-drying, the samples were stored at −30 °C until the experiments were performed. The identification of the sample was accomplished via morphological observation under a microscope by one of the authors (M.F.). *Moorea producens* was a dominant cyanobacteria species in the sample. The sample also contained some unidentified diatoms. The voucher specimen (20100713-a) was deposited at the collection of Marine Natural Products Laboratory of Tokyo University of Marine Science and Technology.

### 3.3. Extraction and Isolation

A frozen sample of the cyanobacterium *M. producens* (dry weight: 0.87 kg) was soaked for several days in ethanol at room temperature. After filtration of the ethanol extract, the sample was extracted five times with methanol and once with acetone. The extracts were then combined and concentrated in vacuo to yield a residue (37.8 g), which was partitioned between methanol/water (4:1, *v*/*v*) and hexane. The 80% MeOH-soluble layer was concentrated to dryness, and the remaining sample was partitioned between distilled water and ethyl acetate (EtOAc). The EtOAc layer was evaporated to dryness. The distilled water layer was then dissolved with 1-butanol (BuOH) and separated into two extracts. Since the EtOAc layer of the extracts from the cyanobacterium *M. producens* showed the most potent bioactivity, this layer was separated using an open glass column (PEGASIL ODS, Senshu Co., Tokyo, Japan) measuring 20 × 120 mm with stepwise elution in 50%, 70%, 90%, and 100% methanol. The 70% methanol eluate was then purified via HPLC using a reverse-phase column (Cosmosil 5C18-AR-II, 10 × 250 mm, Nakalai Tesque Inc., Kyoto, Japan, solvent: 50% methanol, flow rate: 2.0 mL/min, UV: 210 nm). Finally, neo-aplysiatoxin A (**1**, 0.54 mg), neo-debromoaplysiatoxin A (**2**, 1.75 mg), dolastatin 3 (**3**, 3.93 mg), lyngbic acid (**4**, 2.16 mg), malyngamide M (**5**, 0.89 mg), hermitamide A (**6**, 1.01 mg), (−)-loliolide (**7**, 2.79 mg) and (+)-epiloliolide (**8**, 0.81 mg) were isolated.

### 3.4. Biological Tests

Cytotoxicity assays against mouse L1210 leukemia cells were carried out for the isolated compounds. The growth inhibition activities of the compounds (**1**–**8**) against the marine diatom *Nitzschia amabilis* were also evaluated. Both types of bioactive assays were performed using the XTT colorimetric reaction method, as previously reported [30,31].

## Figures and Tables

**Table 1:** NMR data for neo-aplysiatoxin A (**1**) in methanol-*d*_4_ (600 MHz for ^1^H and 150 MHz for ^13^C).

No.	δ_H_ Multip. (*J* in Hz)	δ_C_	No.	δ_H_ Multip. (*J* in Hz)	δ_C_
1		168.9, C	16		142.8, C
2	4.47 s	55.6, CH	17		111.6, C
3		203.3, C	18	7.35 d (8.7)	132.9, CH
4		74.4, C	19	6.65 dd (8.6, 3.1)	115.9, CH
5a	1.62 d (14.5)	48.5, CH_2_	20		157.5, C
5b	2.09 d (14.5)		21	6.95 d (3.0)	114.0, CH
6		41.6, C	22	0.83 (3H) d (3.3)	10.5, CH_3_
7		83.1, C	23	0.84 (3H) d (3.6)	12.2, CH_3_
8a	2.05 dd (15.9, 4.5)	30.7, CH_2_	24	1.33 (3H) s	23.5, CH_3_
8b	2.19 dd (15.9, 4.5)		25	1.01 (3H) s	24.5, CH_3_
9	5.01 m	71.8, CH	26	1.27 (3H) s	24.0, CH_3_
10	1.67 m	32.9, CH	27		171.0, C
11	4.22 dd (10.8, 1.7)	73.8, CH	28a	2.67 dd (13.5, 4.3)	34.3, CH_2_
12	1.58 m	34.2, CH	28b	3.13 dd (13.5, 8.2)	
13a	1.45 m	30.4, CH_2_	29	5.10 ddd (8.2, 5.2, 4.4)	73.5, CH
13b	1.45 m		30	3.93 m	67.4, CH
14a	1.69 m	34.9, CH_2_	31	1.25 (3H) d (6.4)	17.5, CH_3_
14b	1.69 m		32	3.27 (3H) s	56.1, CH_3_
15	4.54 dd (7.5, 4.5)	82.6, CH			

**Table 2:** Inhibition rates on cytotoxicity and diatom growth inhibition assays.

Compound	Cytotoxicity (%)	Diatom Growth Inhibition (%)
**1**	85	90
**2**	85	85
**3**	0	0
**4**	0	0
**5**	90	55
**6**	20	90
**7**	0	0
**8**	0	0

L1210 mouse lymphoma cells were used for the cytotoxicity assay. A marine diatom *Nitzschia amabilis* was used for the diatom growth inhibition assay. The values indicate inhibition rates at the sample concentration of 10 µg/mL.

## Supplementary Materials

The following are available online at https://www.mdpi.com/1420-3049/25/3/457/s1, Figure S1: ESI-HRMS spectrum of neo-aplysiatoxin A in negative ion mode. Figure S2: ^1^H NMR spectrum of neo-aplysiatoxin A in methanol-*d*_4_. Figure S3: ^13^C NMR spectrum of neo-aplysiatoxin A in methanol-*d*_4_. Figure S4: ^1^H–^1^H COSY NMR spectrum of neo-aplysiatoxin A in methanol-*d*_4_. Figure S5: ^1^H–^13^C HSQC spectrum of neo-aplysiatoxin A in methanol-*d*_4_. Figure S6: ^1^H–^13^C HMBC spectrum of neo-aplysiatoxin A in methanol-*d*_4,_ Figure S7. NOESY spectrum of neo-aplysiatoxin A in methanol-*d*_4_.
