# Supplementary material for: Neo-Aplysiatoxin A Isolated from Okinawan Cyanobacterium Moorea Producens
Source: Molecules. 2020 Jan 22;25(3):457. doi: 10.3390/molecules25030457 (PMC7037229; doi:10.3390/molecules25030457)
Supplement: Supplementary file 1 [file molecules-25-00457-s001.pdf]

Supplementary Materials:

Neo-aplysiatoxin A; a new aplysiatoxin derivative from Okinawan cyanobacterium *Moorea producens*

Mioko Kawaguchi, Masayuki Satake, Bo-Tao Zhang, Yue-Yun Xiao, Masayuki Fukuoka, Hajime Uchida, Hiroshi Nagai

Figure S1. ESI-HRMS spectrum of neo-aplysiatoxin A in negative ion mode
Figure S2. ^{1}H NMR spectrum of neo-aplysiatoxin A in methanol-d_4
Figure S3. ^{13}C NMR spectrum of neo-aplysiatoxin A in methanol-d_4
Figure S4. ^{1}H–^{1}H COSY NMR spectrum of neo-aplysiatoxin A in methanol-d_4
Figure S5. ^{1}H–^{13}C HSQC spectrum of neo-aplysiatoxin A in methanol-d_4
Figure S6. ^{1}H–^{13}C HMBC spectrum of neo-aplysiatoxin A in methanol-d_4
Figure S7. NOESY spectrum of neo-aplysiatoxin A in methanol-d_4

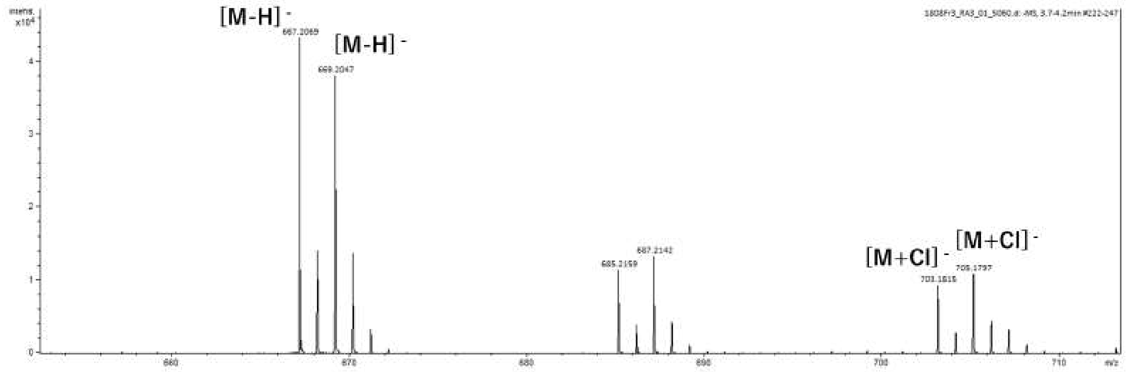

Figure S1. ESI-HRMS spectrum of neo-aplysiatoxin A in negative ion mode

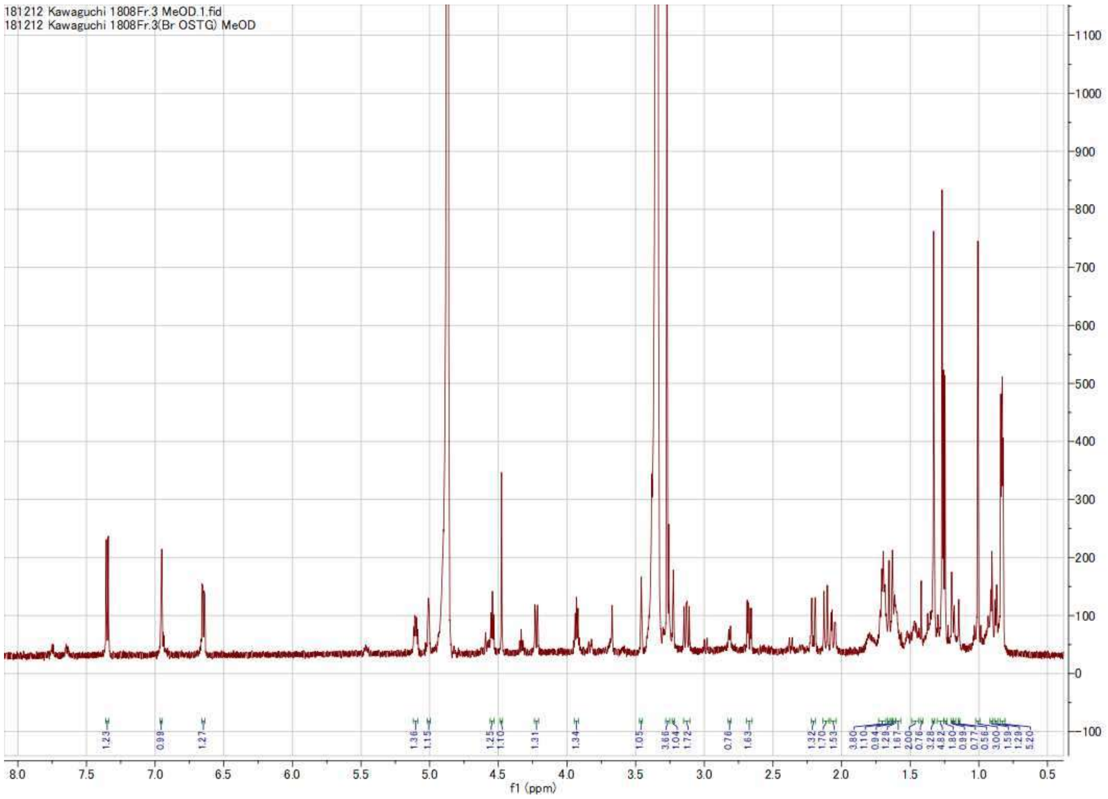

Figure S2. ^{1}H NMR spectrum of neo-aplysiatoxin A in methanol-d_4

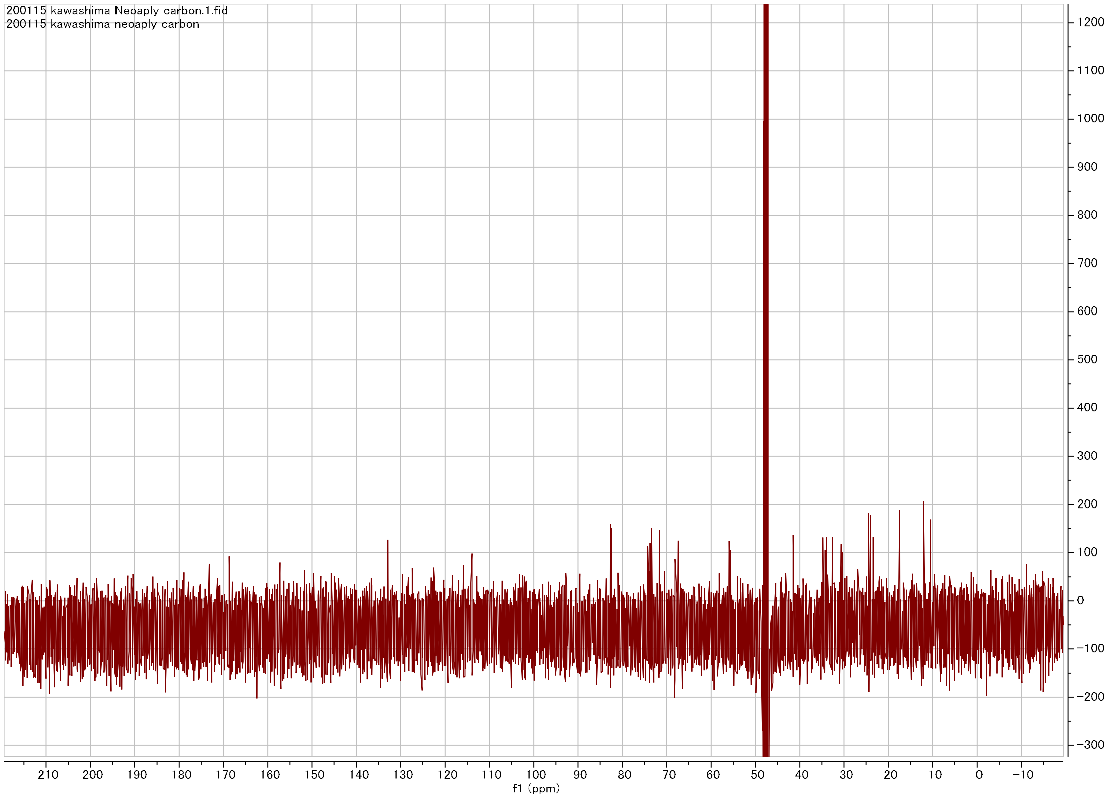

Figure S3. ^{13}C NMR spectrum of neo-aplysiatoxin A in methanol-d_4

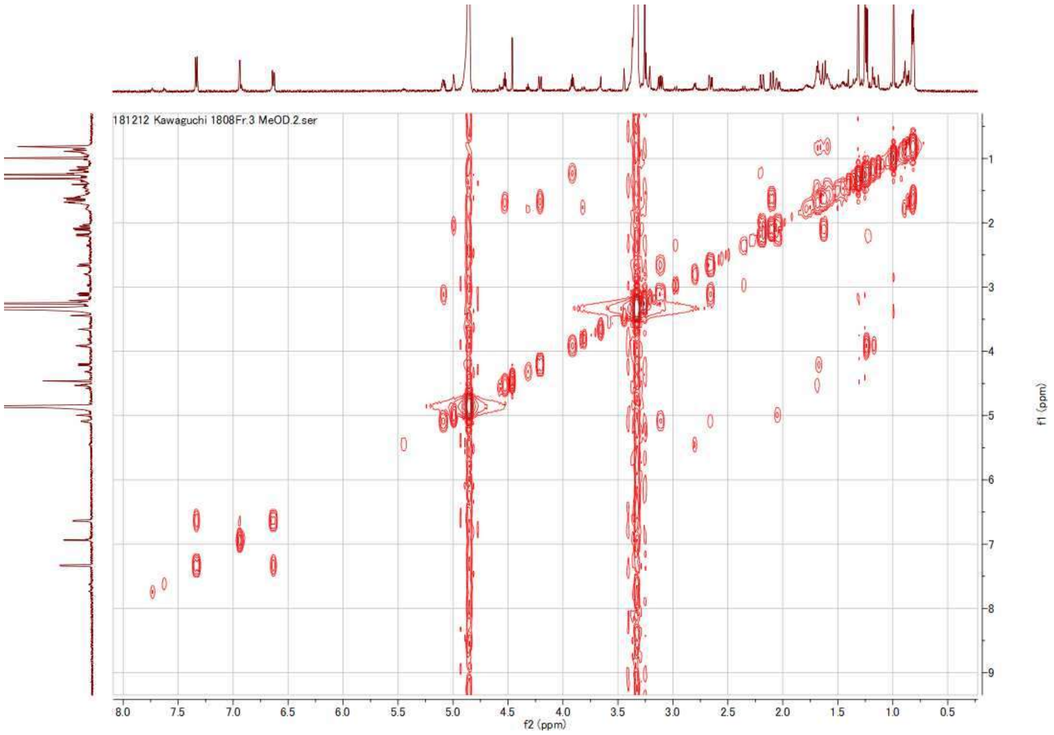

Figure S4. ^{1}H–^{1}H COSY NMR spectrum of neo-aplysiatoxin A in methanol-d_4

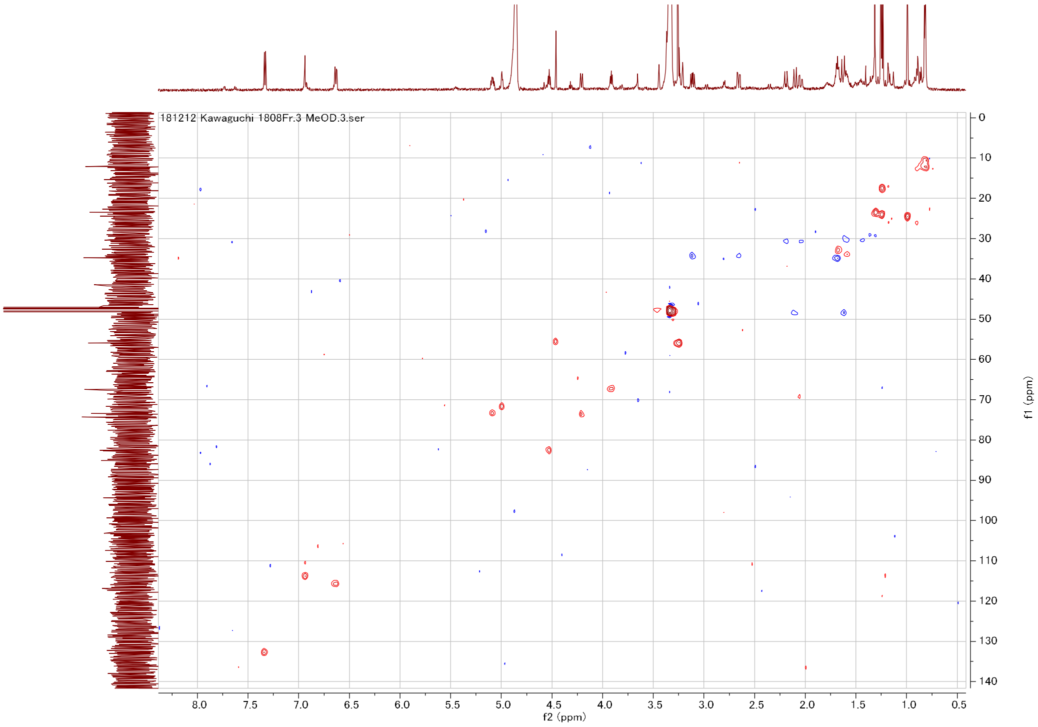

Figure S5. ^{1}H–^{13}C HSQC spectrum of neo-aplysiatoxin A in methanol-d_4

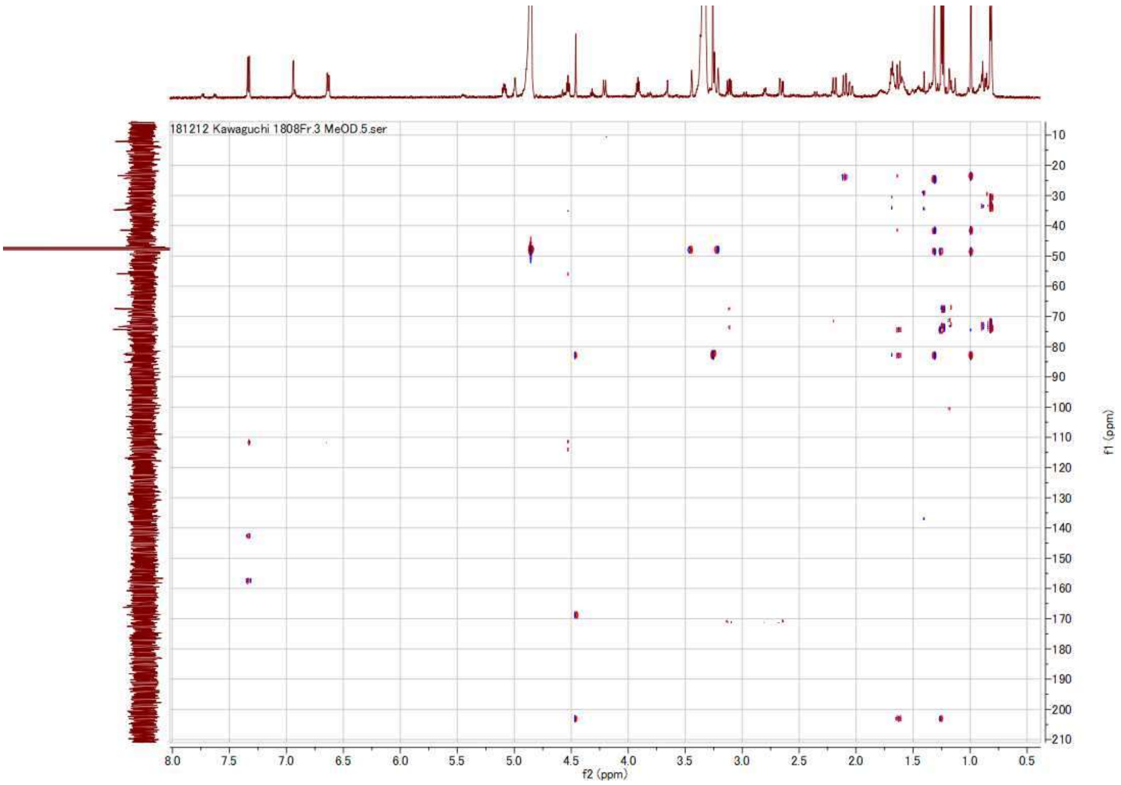

Figure S6. ^{1}H–^{13}C HMBC spectrum of neo-aplysiatoxin A in methanol-d_4

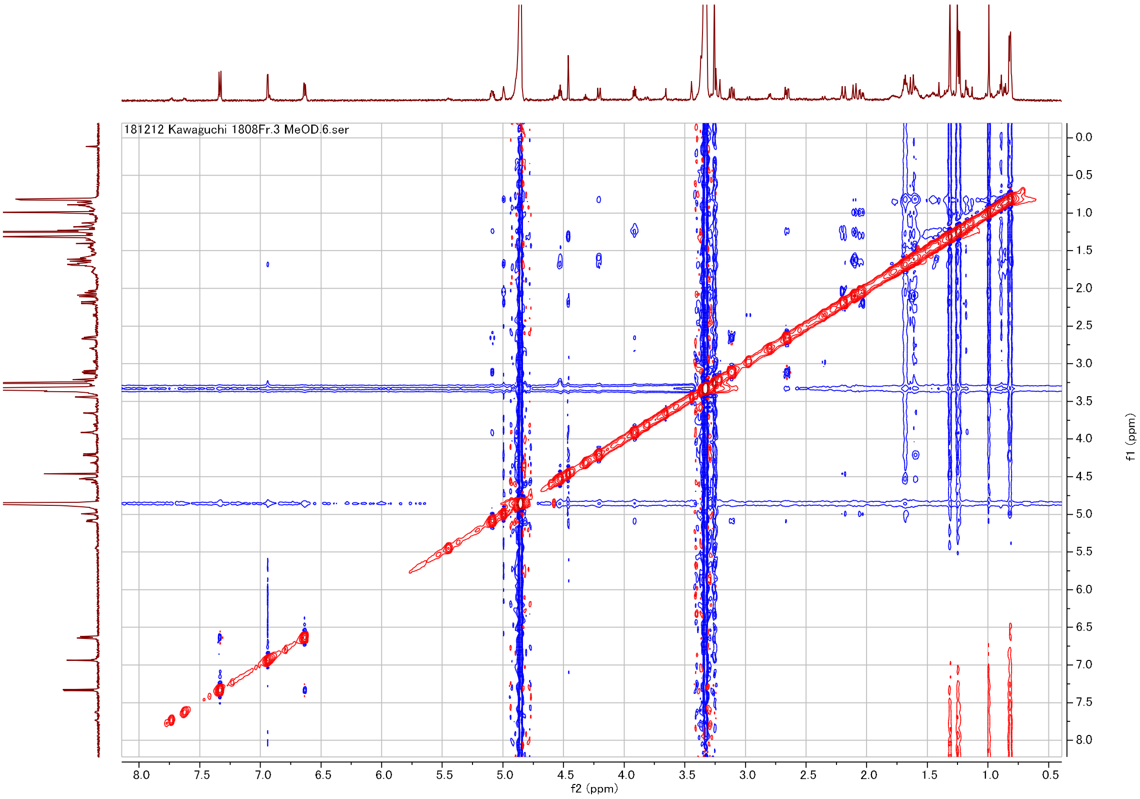

Figure S7. NOESY spectrum of neo-aplysiatoxin A in methanol-d_4
